# Multiplexed Immunoassay Panel Identifies Novel CSF Biomarkers for Alzheimer's Disease Diagnosis and Prognosis

**DOI:** 10.1371/journal.pone.0018850

**Published:** 2011-04-19

**Authors:** Rebecca Craig-Schapiro, Max Kuhn, Chengjie Xiong, Eve H. Pickering, Jingxia Liu, Thomas P. Misko, Richard J. Perrin, Kelly R. Bales, Holly Soares, Anne M. Fagan, David M. Holtzman

**Affiliations:** 1 Department of Neurology, Washington University School of Medicine, St. Louis, Missouri, United States of America; 2 The Knight Alzheimer's Disease Research Center, Washington University School of Medicine, St. Louis, Missouri, United States of America; 3 Division of Biostatistics, Washington University School of Medicine, St. Louis, Missouri, United States of America; 4 Division of Neuropathology, Washington University School of Medicine, St. Louis, Missouri, United States of America; 5 Department of Pathology and Immunology, Washington University School of Medicine, St. Louis, Missouri, United States of America; 6 Hope Center for Neurological Disorders, Washington University School of Medicine, St. Louis, Missouri, United States of America; 7 Neuroscience Research Unit, Pfizer Global Research and Development, Groton, Connecticut, United States of America; 8 Neuroscience Research Unit, Pfizer Global Research and Development, St. Louis, Missouri, United States of America; Mental Health Research Institute of Victoria, Australia

## Abstract

**Background:**

Clinicopathological studies suggest that Alzheimer's disease (AD) pathology begins ∼10–15 years before the resulting cognitive impairment draws medical attention. Biomarkers that can detect AD pathology in its early stages and predict dementia onset would, therefore, be invaluable for patient care and efficient clinical trial design. We utilized a targeted proteomics approach to discover novel cerebrospinal fluid (CSF) biomarkers that can augment the diagnostic and prognostic accuracy of current leading CSF biomarkers (Aβ42, tau, p-tau181).

**Methods and Findings:**

Using a multiplexed Luminex platform, 190 analytes were measured in 333 CSF samples from cognitively normal (Clinical Dementia Rating [CDR] 0), very mildly demented (CDR 0.5), and mildly demented (CDR 1) individuals. Mean levels of 37 analytes (12 after Bonferroni correction) were found to differ between CDR 0 and CDR>0 groups. Receiver-operating characteristic curve analyses revealed that small combinations of a subset of these markers (cystatin C, VEGF, TRAIL-R3, PAI-1, PP, NT-proBNP, MMP-10, MIF, GRO-α, fibrinogen, FAS, eotaxin-3) enhanced the ability of the best-performing established CSF biomarker, the tau/Aβ42 ratio, to discriminate CDR>0 from CDR 0 individuals. Multiple machine learning algorithms likewise showed that the novel biomarker panels improved the diagnostic performance of the current leading biomarkers. Importantly, most of the markers that best discriminated CDR 0 from CDR>0 individuals in the more targeted ROC analyses were also identified as top predictors in the machine learning models, reconfirming their potential as biomarkers for early-stage AD. Cox proportional hazards models demonstrated that an optimal panel of markers for predicting risk of developing cognitive impairment (CDR 0 to CDR>0 conversion) consisted of calbindin, Aβ42, and age.

**Conclusions/Significance:**

Using a targeted proteomic screen, we identified novel candidate biomarkers that complement the best current CSF biomarkers for distinguishing very mildly/mildly demented from cognitively normal individuals. Additionally, we identified a novel biomarker (calbindin) with significant prognostic potential.

## Introduction

With the growing prevalence of Alzheimer's disease (AD), the ability to accurately and reliably diagnose AD in its earliest stages has become a public health priority. The concept of ‘earliest stages,’ however, warrants revision as it is increasingly clear there exists a ‘preclinical’ or ‘presymptomatic’ stage during which the pathological changes associated with AD, amyloid plaques, neurofibrillary tangles, and neuroinflammation, begin to appear without concomitant clinical features. This period has been estimated to be ∼10–15 years in duration. Means to identify this preclinical phase of AD may facilitate medical intervention to prevent or slow neurodegeneration and the resulting cognitive impairment. Because clinical examination cannot detect preclinical disease and is less accurate with very mild stages of AD, there is a pressing need for biomarkers for AD. Furthermore, biomarkers may have significant utility in clinical trial design, providing greater diagnostic certainty for enrollment than is possible by clinical diagnosis alone, and allowing for the selective enrollment of individuals at greater risk of developing future cognitive impairment, ultimately resulting in trials of shorter duration, smaller size, and reduced cost.

The cerebrospinal fluid (CSF) is a logical source of potential AD biomarkers, as it reflects biochemical changes in the brain. Indeed, the fluid biomarkers thus far showing the greatest promise for use in AD diagnosis and prognosis are CSF amyloid-β42 (Aβ42), tau, and phosphorylated forms of tau (p-tau) [1]]–[[5]. Concentrations of CSF Aβ42 decrease in association with the deposition of Aβ42 into plaques within the brain [6]]–[[9]. This process occurs years prior to the clinical onset of AD and may mark the earliest phase of AD pathology. CSF Aβ42 levels remain low throughout the disease course [6], [10], [11]. In contrast, CSF tau and p-tau levels progressively increase with the advancing stages of AD, and in some individuals, begin to rise several years prior to diagnosis [7], [12], [13]. The ratios of tau or p-tau to Aβ42 have also proven useful for predicting clinical progression in individuals who have very mild dementia or mild cognitive impairment (MCI), and, importantly, for predicting future MCI and AD dementia among those who are cognitively normal [7], [14], [15]. Nevertheless, even for these analytes, there is substantial overlap between control and AD groups and a need for better prognostic ability [16]. Consequently, there remains a need for supplemental biomarkers to improve diagnosis and prognosis at different disease stages. Given the multifactorial nature of AD pathophysiology, it is likely that there will be other CSF biomarkers that will be useful in this regard. While proteomic screens have identified a number of other candidate AD biomarkers [17]]–[[26], few studies have utilized large, well-characterized cohorts or have looked for biomarkers in preclinical or very early stage disease.

In this study, a large number of CSF samples (N = 333) selected from well-characterized MCI/very early stage-AD and cognitively normal control cohorts were chosen for protein profiling using a commercially available panel that measures a variety of cytokines, chemokines, metabolic markers, growth factors, and other markers. Multiplex immunoassay platforms such as the one used here, Rules Based Medicine Discovery MAP 1.0 panel, allow for the simultaneous quantitation of many analytes, and by adhering to clinical laboratory improvement amendments (CLIA) standards, are amenable for clinical trial work. Using multiple statistical approaches, we have identified a set of novel biomarkers that may improve the ability of traditional AD biomarkers, Aβ42 and tau, to distinguish MCI/early-stage AD from cognitive normalcy and to predict the development of future cognitive impairment (i.e. detection of preclinical AD at increased risk of progression).

## Methods

### Ethics Statement

The study protocols were approved by the Human Studies Committees at all participating institutions, and written and verbal informed consent was obtained from participants at enrollment. All aspects of this study were conducted according to the principles expressed in the Declaration of Helsinki.

### Participant Selection

Participants (N = 333) were community-dwelling volunteers enrolled in longitudinal studies of healthy aging and dementia at the Knight Alzheimer's Disease Research Center at Washington University (WU-ADRC). The study protocol was approved by the Human Studies Committee at Washington University, and written and verbal informed consent was obtained from participants at enrollment. At sample collection, participants were ≥60 years of age and in good general health, having no other neurological, psychiatric, or major medical diagnoses that could contribute importantly to dementia. Clinical diagnosis was evaluated based on criteria from the National Institute of Neurological and Communicative Diseases and Stroke-Alzheimer's Disease and Related Disorders Association (NINCDS-ADRDA) [27]. Cognitive status was rated with the clinical dementia rating scale (CDR); a CDR of 0 (N = 242) indicated no dementia, CDR 0.5 (N = 63) indicated very mild dementia, and CDR 1 (N = 28) indicated mild dementia [28]. Some of the CDR 0.5 study participants met the criteria for mild cognitive impairment (MCI) and some were more mildly impaired and were considered “pre-MCI” [29], [30]. A subset of participants (N = 179) in this cohort had also undergone positron emission tomography (PET) imaging with Pittsburgh Compound-B (PIB) for assessment of in vivo amyloid burden [32]. A mean cortical PIB binding potential value was obtained by averaging prefrontal cortex, precuneus, lateral temporal cortex, and gyrus rectus regions, as described previously [6], [31]. Apolipoprotein E (*APOE*) genotype was determined by the WU-ADRC Genetics Core. Twenty-five to 30 mL of CSF was collected by lumbar puncture (LP) at 8 AM following overnight fasting. Samples were inverted to avoid gradient effects, centrifuged briefly (2,000g, 5 minutes, 4°C) to remove any cellular elements, and aliquoted into polypropylene tubes for freezing and storage at −80°C [7].

### Analyte Measurements

CSF Aβ42, total tau, and phospho-tau181 levels (henceforth referred to as ‘traditional’ biomarkers) were analyzed in duplicate by the WU-ADRC Biomarker Core by quantitative ELISA after a single freeze-thaw cycle according to the manufacturer's specifications (Innotest, Innogenetics, Ghent, Belgium).

CSF samples were also evaluated by Rules Based Medicine, Inc. (RBM) (Austin, TX) for levels of 190 analytes using the Human Discovery Multi-Analyte Profile (MAP) 1.0 panel and a Luminex 100 platform. This 190 analyte panel (from here on referred to as ‘RBM analytes’) was assembled by RBM to measure a range of cytokines, chemokines, growth factors, hormones, metabolic markers, and other proteins thought to be important in disease; a complete list of analytes is available at www.rulesbasedmedicine.com.

At RBM, the samples were thawed at room temperature (RT), vortexed, spun at 13,000g for 5 minutes for clarification, and 1.0 mL was removed into a master microtiter plate for MAP analysis. Using automated pipetting, an aliquot of each sample was introduced into one of the capture microsphere multiplexes of the Human DiscoveryMAP. The mixtures of sample and capture microspheres were thoroughly mixed and incubated at RT for 1 hour. Multiplexed cocktails of biotinylated reporter antibodies for each multiplex were then added robotically, and after thorough mixing, were incubated for an additional hour at RT. Multiplexes were developed using an excess of streptavidin-phycoerythrin solution which was thoroughly mixed into each multiplex and incubated for 1 hour at RT. The volume of each multiplexed reaction was reduced by vacuum filtration and then increased by dilution into matrix buffer for analysis. Analysis was performed in a Luminex 100 instrument and the resulting data stream was interpreted using proprietary data analysis software developed at RBM. For each multiplex, both calibrators and controls were included on each microtiter plate. Eight-point calibrators were run in the first and last column of each plate and 3-level quality controls were included in duplicate. Testing results were determined first for the high, medium and low controls for each multiplex to ensure proper assay performance. Unknown values for each of the analytes localized in a specific multiplex were determined using 4 and 5 parameter, weighted and non-weighted curve fitting algorithms included in the data analysis package.

### Statistical Analysis

Statistical analyses were performed in SAS 9.2 (SAS Institute Inc, Cary, NC) for univariate analyses, ROC/AUC calculations, and Cox proportional hazards models, and in R version 2.10.1 for predictive modeling [packages/versions: caret (4.65), earth (2.4-0), kernlab (0.9–9), klaR (0.6–3), MASS (7.3–7), mda (0.4–1), nnet (7.3–1), pamr (1.44.0), pls (2.1–0), randomForest (4.5–34), spls (2.1–0)] [33]. Of the 190 RBM analytes, 65 had >10% of data missing or below the lower detection limit (LDL), and were therefore excluded from analysis, yielding 125 ‘measurable’ analytes. Data below the LDL were imputed to LDL/2, and data more than five standard deviations beyond the mean were imputed using a nearest neighbor algorithm. Of the 125 measurable analytes, 24 analytes had at least one value below the LDL, imputed to LDL/2. For those 24 analytes, the percentage of data imputed ranged from < 1% (3 or fewer values) to 9.5% (33 values). There were a total of 82 outliers from 48 participants, with outliers in a maximum of 10 analytes for one participant, and in 2 – 9 analytes for the remaining participants. The distributions of analytes were tested for normality by Box-Cox analysis and, when appropriate, log10 transformed to approximate a normal distribution. Correlations between RBM analytes, traditional AD biomarkers, and demographic variables were evaluated using the Spearman rho correlation coefficient (α = 0.05). Analysis of covariance (ANCOVA) using the General Linear Model (GLM) procedure in SAS was used to determine analytes that differed in concentration between AD and control groups while adjusting for the effects of age and gender. Bonferroni correction was used to adjust for multiple testing (128 RBM plus traditional analytes). For each analyte showing promise by univariate analysis, the area under the Receiver Operating Characteristic (ROC) curve (AUC) was calculated for discriminating CDR 0 versus CDR>0. The method of Xiong et al. [34] was implemented to determine the optimum linear combination of analytes and to calculate the confidence interval (CI) on the AUC and the sensitivity. A bootstrapping resampling technique was used to obtain robust estimates of expected future performance of the three marker panels in predicting CDR 0 versus CDR>0. Averages of performance measures (the 95% CI of the AUC, sensitivity at 80% specificity, and p-value) were taken over 100 iterations of the bootstrap.

Cox proportional hazard models assessed the ability of baseline biomarkers to predict conversion from cognitive normalcy (CDR 0) to very mild or mild dementia (CDR 0.5 and 1). Data from participants who did not convert during the follow-up were statistically censored at the date of last assessment. Biomarker measurements were treated as continuous variables and were converted to standard Z-scores. Baseline variables were considered for inclusion in multivariate models if they were associated with time to conversion in a univariate analysis (p<0.15). Variables were retained in multivariate proportional hazard models if p<0.05. AIC (Akaike Information Criterion), a measure of goodness of fit of an estimated statistical model, was used to compare different models, with a lower AIC indicating better model fit.

Several statistical machine learning techniques were utilized to predict CDR status as a function of baseline characteristics (e.g. age) and the candidate biomarkers. Rather than focusing on a specific model, a panel of predictive modeling techniques was applied to the data. Most of these models contain “tuning parameters” that cannot be directly estimated from the data; these values were chosen using resampling techniques. The models used were:

- Partial Least Squares (PLS) is a latent variable model that produces linear class boundaries and works well with correlated predictors [35]. Candidate values of the tuning parameter, the number of PLS components, ranged from 1 to 20.
- Sparse Partial Least Squares (SPLS) is a variant of PLS that incorporates feature selection in the model fitting [36]. The number of PLS components was varied in the same manner as the basic PLS model and the additional tuning parameter for regularization was varied from 0.1 to 0.9.
- Random Forests (RF) is a tree-based ensemble method [37]. The number of randomly selected variables at each split was varied over five values (generally 2 to the number of predictors in the model).
- Boosted Trees are another tree-based ensemble model [38]. The three tuning parameters are the depth of the tree (even values from 2 to 10 were evaluated), the number of boosting iterations (20 iterations to 2000 in 100 iteration increments) and the learning rate (fixed at 0.1).
- Support Vector Machine (SVM) are a kernel based method [39]. The radial basis function kernel was used. The kernel parameter was estimated analytically [40] and the five candidate values of the cost parameter ranged from 0.1 to 1,000 on the log10 scale.
- Nearest Shrunken Centroids (NCS) is a prototype model that incorporates feature selection [41]. The tuning parameter, the shrinkage threshold, was varied over 30 values (between 0.325 and 9.097 for the model using traditional biomarkers, and between 0.325 and 9.11 for the model using traditional and RBM markers.)
- Naïve Bayes (NB) is a simple classifier where each predictor variable contributes to the final class prediction independently [42]. The conditional distributions were computed using a simple Gaussian distribution or using a nonparametric density estimator.
- K-Nearest Neighbors (KNN) is a simple prototype based model [42]. Candidate values for the number of neighbors ranged from 5 to 15.
- Flexible Discriminant Analysis (FDA) is a partitioning based model that also incorporates feature selection [43]. The multivariate adaptive spline basis function was used. Ten candidate values for the number of retained terms were evaluated.

To determine the values for the tuning parameters and to estimate performance, resampling methods were used. The entire data set was repeatedly split into training (80%) and test sets (20%). This process was repeated 200 times. Models were fit on the training sets and the associated held-out values were used to estimate performance (sensitivity, specificity, and the area under the ROC curve). The final estimates of performance were calculated by averaging the 200 sets of performance values from the resampling procedure.

## Results

### Levels of 37 markers are altered in MCI/very mild and mild AD CSF

To identify new candidate biomarkers for AD, multiplexed Luminex-based immunoassays were used to evaluate the levels of 190 analytes in the CSF of 242 cognitively normal participants (CDR 0), 63 participants with very mild dementia (CDR 0.5), and 28 participants with mild dementia (CDR 1) (participant characteristics at baseline assessment in Table 1). Since the number of CDR 1 participants was relatively smaller, and all CDR 0.5 and CDR 1 participants were clinically diagnosed as having AD, the CDR 0.5 and CDR 1 groups were combined in the statistical analyses. There were no statistically significant differences in age, gender, MCBP for PIB-PET, or *APOE* genotype between the CDR 0.5 and CDR 1 groups. Of the 125 RBM analytes that were statistically assessed (Table S1), the mean concentrations of 37 CSF analytes were found to differ between cognitively normal (CDR 0) and very mildly/mildly demented (CDR 0.5 and 1) participants by analysis of covariance (ANCOVA) adjusting for age and gender (p<0.05) (Table 2 and Table S2). Twelve of these 37 analytes remained significant after Bonferroni correction for multiple testing (n = 128, adjusted alpha  = 0.0004). Additionally, participants with very mild/mild dementia exhibited the typical AD CSF biomarker profile characterized by significantly lower mean levels of CSF Aβ42 and higher mean levels of CSF tau and CSF p-tau181, as well as displaying higher mean cortical amyloid burden (MCBP assessed by PIB-PET imaging) as has been seen previously (Tables 1 and 2) [6], [31], [32].

**Table 1:** Demographic, clinical, and genotypic characteristics of the 333 study participants.

Characteristic	CDR 0	CDR 0.5	CDR 1
N	242	63	28
Gender (% Female)	65%	52%	50%
*APOE* genotype, % ε4+	32%	54%	57%
Mean MMSE score (SD)	28.9 (1.3)	26.3 (2.8)	22.5 (4.0)
Mean age at LP (SD), yrs	71.6 (7.4)	74.6 (7.3)	76.8 (6.2)
Mean CSF Aβ42 (SD), pg/mL	607 (234)	436 (233)	355 (119)
Mean CSF tau (SD), pg/mL	315 (169)	547 (278)	557 (266)
Mean CSF p-tau181 (SD), pg/mL	56 (25)	85 (45)	78 (38)
Mean PIB MCBP (SD)	0.12 (0.24)	0.54 (0.34)	0.50 (0.50)

Abbreviations: CDR, Clinical Dementia Rating; *APOE*, apolipoprotein E; MMSE, Mini-Mental State Examination; LP, lumbar puncture; SD, standard deviation; CSF, cerebrospinal fluid; Aβ-42, amyloid-beta peptide 1-42; p-tau181, tau phosphorylated at threonine 181; PIB MCBP, Pittsburgh Compound B mean cortical PIB binding potential. MCBP data available for 179 study participants.

**Table 2:** Analytes that differ in levels between cognitively normal (CDR 0) and very mildly/mildly demented (CDR 0.5 and 1) participants.

Marker	Adjusted mean CDR 0	Adjusted mean CDR>0	p	Raw mean CDR 0	Raw mean CDR>0
Aβ42 (pg/mL)*	607.45	418.85	<0.0001	606.90	411.18
Tau (pg/mL)*	315.59	533.60	<0.0001	314.80	549.96
p-tau181 (pg/mL)*	56.30	81.01	<0.0001	56.32	82.98
Growth-Regulated alpha protein (GRO-α) (pg/mL)*	18.27	22.09	<0.0001	18.30	22.44
Log Matrix Metalloproteinase-10 (MMP-10) (pg/mL)*	24.84	31.41	<0.0001	24.11	32.61
Log N-terminal pro-brain natriuretic peptide (NT-proBNP) (pg/mL)*	87.00	107.75	<0.0001	87.70	111.12
Log Plasminogen Activator Inhibitor 1 (PAI-1) (ng/mL)*	1.05	1.28	<0.0001	1.01	1.34
TNF-Related Apoptosis-Inducing Ligand Receptor 3 (TRAIL-R3) (ng/mL)*	0.55	0.63	<0.0001	0.55	0.65
Vascular Endothelial Growth Factor (VEGF) (pg/mL)*	441.57	378.30	<0.0001	437.83	386.01
Log Pancreatic Polypeptide (PP) (pg/mL)*	0.94	1.30	0.0001	0.88	1.41
Log FAS (ng/mL)*	0.57	0.65	0.0002	0.56	0.67
Log Macrophage Migration Inhibitory Factor (MIF) (ng/mL)*	0.15	0.17	0.0004	0.15	0.18
Interleukin-7 (IL-7) (pg/mL)	12.63	9.47	0.0006	12.23	9.68
Log Cystatin C (ng/mL)	5613.84	4750.89	0.0011	5551.50	4835.30
Thrombopoietin (ng/mL)	0.43	0.37	0.0016	0.42	0.37
Sortilin (ng/mL)	6.32	6.92	0.0019	6.33	6.96
Monocyte Chemotactic Protein 2 (MCP-2) (pg/mL)	4.03	4.61	0.0020	3.97	4.67
Log Fibrinogen (ug/mL)	0.63	0.78	0.0024	0.59	0.81
Log Creatine Kinase-MB (CKMB) (pg/mL)	26.55	20.97	0.0030	26.62	20.87
Cortisol (ng/mL)	11.21	12.65	0.0034	11.17	12.89
Thymus-Expressed Chemokine (TECK) (ng/mL)	6.38	6.85	0.0039	6.30	6.96
Eotaxin-3 (pg/mL)	56.78	62.09	0.0057	55.33	63.68
Interleukin-17E (IL-17E) (pg/mL)	8.63	7.75	0.0058	8.60	7.79
Kidney Injury Molecule-1 (KIM-1) (pg/mL)	78.97	83.46	0.0074	79.05	83.08
Log Heparin-binding epidermal growth factor-like growth factor (HB-EGF) (pg/mL)	24.98	28.77	0.0077	25.05	28.70
Log Osteopontin (ng/mL)	173.23	197.68	0.0078	174.15	202.31
Log α-1-Antitrypsin (ug/mL)	4.87	5.37	0.0102	4.73	5.49
Fatty Acid Synthase Ligand (FASL) (pg/mL)	4.85	5.40	0.0109	4.78	5.49
Log Insulin-like Growth Factor-Binding Protein 2 (IGFBP-2) (ng/mL)	199.58	212.16	0.0111	195.93	217.47
Log Interleukin-10 (IL-10) (pg/mL)	1.14	1.29	0.0131	1.12	1.29
Log Tumor necrosis factor-a receptor 2 (TNF RII) (ng/mL)	0.53	0.59	0.0141	0.52	0.62
Log Resistin (pg/mL)	26.28	30.76	0.0146	25.20	32.14
Log Fatty Acid Binding Protein (FABP) (ng/mL)	3.03	3.62	0.0209	2.93	3.81
Log Apolipoprotein D (ApoD) (ug/mL)	4.18	4.57	0.0318	4.02	4.65
Log Hepatocyte Growth Factor (HGF) (ng/mL)	1.18	1.28	0.0349	1.18	1.30
Log Insulin (uIU/mL)	0.22	0.19	0.0359	0.21	0.19
Log Hemofiltrate cysteine-cysteine chemokine (HCC-4) (pg/mL)	30.25	33.13	0.0418	28.98	33.87
Log Interferon gamma Induced Protein 10 (IP-10) (pg/mL)	299.63	341.86	0.0432	295.14	354.74
Log Gamma-Interferon-Induced Monokine (MIG) (pg/mL)	423.80	493.91	0.0452	400.16	572.75
Thrombomodulin (ng/mL)	0.17	0.18	0.0475	0.17	0.19

Analysis of covariance (ANCOVA) using the General Linear Model (GLM) procedure in SAS was used to determine analytes that differed in concentration (p<0.05) between CDR 0 and CDR>0 groups while adjusting for the effects of age and gender ("adjusted means").

*indicates analytes that were significant after Bonferroni correction based on the number of markers analyzed (128 markers, cutoff of 0.0004 for familywise p<0.05). For markers that were log transformed to approximate a normal distribution, the resulting Least Squares mean (or estimated marginal mean) was back-transformed to obtain the adjusted mean shown. Also provided are the raw mean concentrations for the CDR 0 and CDR>0 groups.

### Correlation of RBM analytes with demographic features and other biomarker values

Because the CDR 0, 0.5, and 1 groups showed somewhat different distributions with regard to age at lumbar puncture and gender, levels of the 37 RBM analytes were evaluated for correlation with these variables. Many analytes were significantly associated with age or gender (Table 3). Additionally, seeking insight into the potential roles of the analytes in AD pathology, we evaluated their association with CSF Aβ42, tau, and p-tau181, and cortical amyloid burden measured by PIB-PET imaging. Many of the analytes correlated with CSF tau and CSF p-tau181 (31 and 30 analytes, respectively), while fewer correlated with CSF Aβ42 or cortical amyloid burden (8 and 5 analytes, respectively) (Table 3).

**Table 3:** Correlations of RBM analytes with age, gender, and other biomarker values.

Analyte	Gender	Age	Aβ42	Tau	p-tau181	tau/Aβ42	Cortical PIB
**α1A**	<0.001	0.255 (<0.0001)	0.031 (0.574)	0.117 (0.033)	0.105 (0.055)	0.048 (0.386)	-0.048 (0.525)
**ApoD**	<0.001	0.218 (<0.0001)	0.059 (0.280)	0.222 (<0.0001)	0.216 (<0.0001)	0.113 (0.039)	-0.103 (0.169)
**Calbindin**	0.001	0.196 (<0.001)	0.094 (0.088)	0.476 (<0.0001)	0.500 (<0.0001)	0.294 (<0.0001)	0.122 (0.104)
**CKMB**	0.524	-0.069 (0.211)	0.008 (0.877)	-0.200 (<0.001)	-0.186 (0.001)	-0.148 (0.007)	0.032 (0.673)
**Cortisol**	0.282	0.252 (<0.0001)	-0.051 (0.357)	0.187 (0.001)	0.189 (0.001)	0.159 (0.004)	0.012 (0.875)
**Cystatin C**	0.461	0.093 (0.089)	0.281 (<0.0001)	0.536 (<0.0001)	0.597 (<0.0001)	0.236 (<0.0001)	-0.041 (0.587)
**Eotaxin-3**	<0.001	0.317 (<0.0001)	0.058 (0.289)	0.367 (<0.0001)	0.342 (<0.0001)	0.217 (<0.0001)	0.003 (0.971)
**FABP**	0.031	0.296 (<0.0001)	0.012 (0.833)	0.727 (<0.0001)	0.725 (<0.0001)	0.505 (<0.0001)	0.159 (0.034)
**FAS**	<0.001	0.297 (<0.0001)	0.083 (0.132)	0.491 (<0.0001)	0.470 (<0.0001)	0.288 (<0.0001)	-0.074 (0.326)
**FASL**	0.165	0.192 (<0.001)	-0.060 (0.274)	0.189 (0.001)	0.200 (<0.001)	0.129 (0.018)	-0.020 (0.795)
**Fibrinogen**	<0.001	0.284 (<0.0001)	-0.044 (0.422)	0.192 (<0.001)	0.178 (0.001)	0.145 (0.008)	0.034 (0.652)
**GRO-α**	0.178	0.279 (<0.0001)	-0.105 (0.056)	0.317 (<0.0001)	0.329 (<0.0001)	0.259 (<0.0001)	0.144 (0.054)
**HB-EGF**	0.975	0.017 (0.751)	0.079 (0.151)	0.348 (<0.0001)	0.359 (<0.0001)	0.202 (<0.001)	-0.024 (0.751)
**HCC-4**	<0.001	0.240 (<0.0001)	0.007 (0.895)	0.094 (0.088)	0.037 (0.504)	0.047 (0.388)	-0.095 (0.204)
**HGF**	0.918	0.222 (<0.0001)	0.088 (0.110)	0.619 (<0.0001)	0.639 (<0.0001)	0.386 (<0.0001)	0.004 (0.957)
**IGFBP-2**	<0.001	0.394 (<0.0001)	0.062 (0.262)	0.462 (<0.0001)	0.441 (<0.0001)	0.278 (<0.0001)	0.031 (0.685)
**IL-17E**	0.386	0.032 (0.563)	0.017 (0.760)	0.007 (0.899)	0.049 (0.371)	0.019 (0.725)	-0.101 (0.180)
**IL-7**	0.007	0-.002 (0.976)	0.147 (0.007)	-0.003 (0.961)	0.032 (0.557)	-0.091 (0.096)	-0.227 (0.002)
**IL-10**	<0.001	0.055 (0.313)	-0.026 (0.637)	0.070 (0.205)	0.075 (0.170)	0.053 (0.337)	-0.071 (0.342)
**IP-10**	0.327	0.236 (<0.0001)	0.023 (0.682)	0.249 (<0.0001)	0.282 (<0.0001)	0.147 (0.007)	-0.071 (0.344)
**Insulin**	<0.001	0.094 (0.088)	0.245 (<0.0001)	0.213 (<0.0001)	0.214 (<0.0001)	0.005 (0.921)	-0.190 (0.011)
**KIM-1**	0.636	0-.032 (0.561)	-0.057 (0.301)	-0.239 (<0.0001)	-0.331 (<0.0001)	-0.154 (0.005)	-0.060 (0.427)
**MCP-2**	0.013	0.146 (0.007)	-0.106 (0.053)	0.045 (0.408)	0.059 (0.282)	0.071 (0.199)	-0.011 (0.880)
**MIF**	0.239	0.330 (<0.0001)	-0.007 (0.901)	0.579 (<0.0001)	0.597 (<0.0001)	0.412 (<0.0001)	0.084 (0.264)
**MIG**	0.528	0.603 (<0.0001)	-0.017 (0.762)	0.282 (<0.0001)	0.289 (<0.0001)	0.207 (<0.001)	-0.053 (0.484)
**MMP-10**	0.002	0.325 (<0.0001)	-0.116 (0.034)	0.458 (<0.0001)	0.415 (<0.0001)	0.390 (<0.0001)	0.086 (0.252)
**NT-proBNP**	0.030	0.273 (<0.0001)	0.053 (0.338)	0.331 (<0.0001)	0.323 (<0.0001)	0.188 (0.001)	-0.007 (0.923)
**Osteopontin**	0.137	0.192 (<0.001)	0.030 (0.590)	0.680 (<0.0001)	0.701 (<0.0001)	0.466 (<0.0001)	0.162 (0.030)
**PP**	<.001	0.374 (<0.0001)	-0.072 (0.189)	0.226 (<0.0001)	0.179 (0.001)	0.192 (<0.001)	0.041 (0.586)
**PAI-1**	<.001	0.429 (<0.0001)	-0.064 (0.244)	0.334 (<0.0001)	0.327 (<0.0001)	0.266 (<0.0001)	-0.003 (0.973)
**Resistin**	<.001	0.355 (<0.0001)	0.072 (0.189)	0.255 (<0.0001)	0.198 (<0.0001)	0.120 (0.029)	-0.075 (0.320)
**Sortilin**	0.881	0.135 (0.014)	0.139 (0.011)	0.515 (<0.0001)	0.527 (<0.0001)	0.273 (<0.0001)	-0.003 (0.972)
**TNF RII**	0.205	0.426 (<0.0001)	0.059 (0.282)	0.678 (<0.0001)	0.702 (<0.0001)	0.442 (<0.0001)	0.002 (0.975)
**TRAIL-R3**	0.112	0.413 (<0.0001)	-0.011 (0.837)	0.509 (<0.0001)	0.476 (<0.0001)	0.356 (<0.0001)	0.008 (0.914)
**Thrombomodulin**	<.001	0.193 (<0.001)	0.109 (0.048)	0.215 (<0.0001)	0.205 (<0.001)	0.076 (0.168)	-0.063 (0.406)
**Thrombopoietin**	0.015	0.034 (0.531)	0.194 (<0.001)	-0.016 (0.768)	0.017 (0.758)	-0.130 (0.017)	-0.237 (0.001)
**TECK**	0.015	0.270 (<0.0001)	0.047 (0.389)	0.322 (<0.0001)	0.312 (<0.0001)	0.193 (<0.001)	0.001 (0.992)
**VEGF**	0.651	0.101 (0.065)	0.357 (<0.0001)	0.470 (<0.0001)	0.543 (<0.0001)	0.154 (0.005)	-0.059 (0.429)

Correlations were evaluated using the Spearman rho correlation coefficient (α = 0.05); shown are the *r* and (p value). Gender differences were evaluated by Mann-Whitney test.

### Diagnostic Utility of Candidate Biomarkers

**Figure 1:**
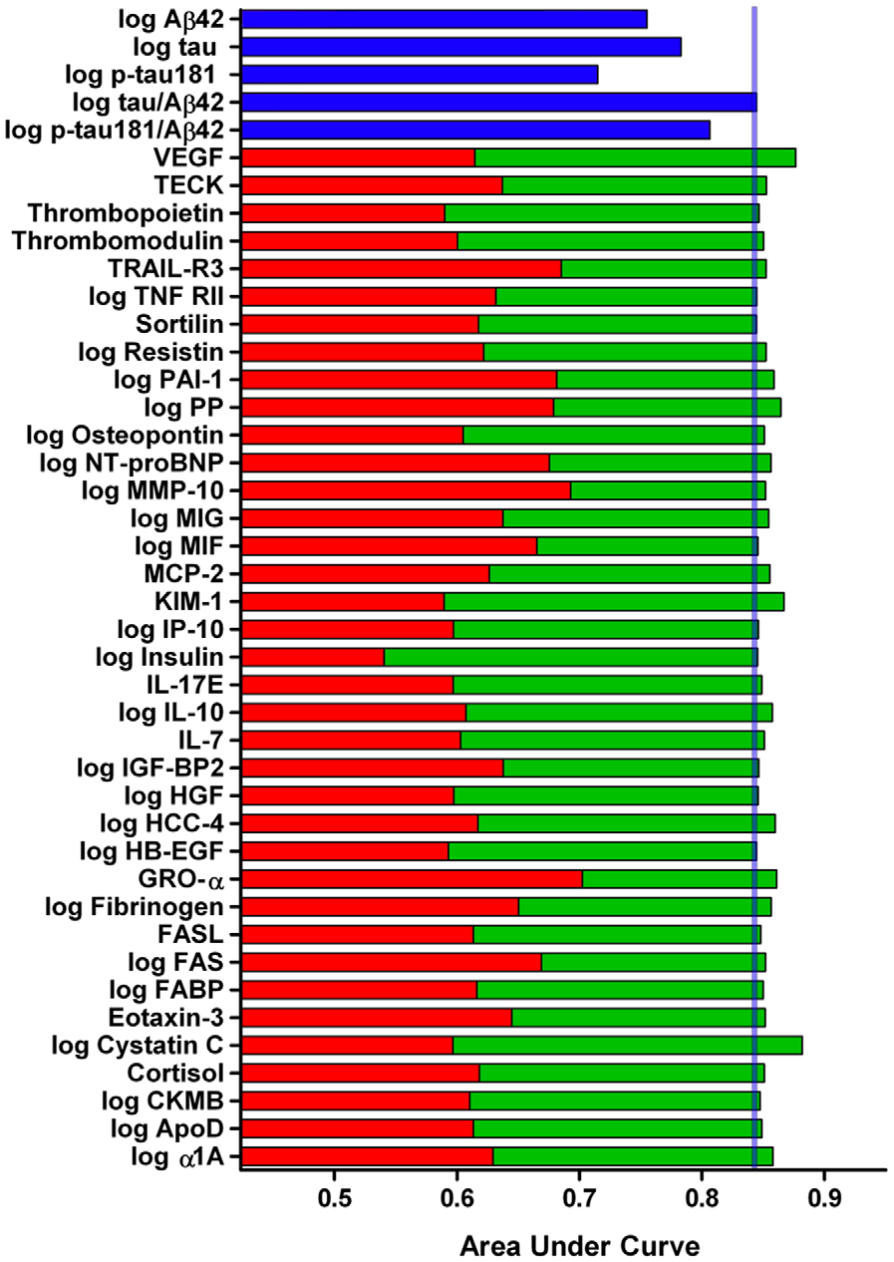
ROC analyses, graphical representation. ROC analyses assessed the ability of the traditional biomarkers (blue) and of the 37 RBM analytes with p<0.05 in the univariate analyses (red) to discriminate CDR>0 from CDR 0 individuals. Combining the best-performing of the traditional biomarkers, the tau/Aβ42 ratio, with RBM analytes improved upon the AUC of tau/Aβ42 in many cases (green).

To assess the potential of the analytes for identifying very mild/mild dementia (combined CDR 0.5 and CDR 1), ROC curves and AUCs were calculated for each of the 37 RBM analytes and for traditional AD biomarkers Aβ42, tau, p-tau181 and the ratios tau/Aβ42 and p-tau181/Aβ42 (Table 4 and Figure 1). Although none of the RBM analytes alone out-performed the traditional biomarkers, combining traditional biomarkers with RBM analytes improved upon the AUC of the traditional biomarkers in many cases; e.g., Aβ42: AUC =  .7552, combinations ranging from .7553–.8201; tau/Aβ42: AUC =  .8443, combinations ranging from .8444–.8819; p-tau181/Aβ42: AUC =  .8065, combinations ranging from .8065–.8468 (Table 4 and Figure 1). For these ‘2-marker panels’ of traditional biomarker plus RBM analyte, combinations with tau/Aβ42 consistently yielded the highest AUCs. To investigate whether combinations of three markers could yield a small panel with improved diagnostic accuracy, we utilized a targeted approach in which the four 2-marker panels with the highest AUCs (tau/Aβ42 + cystatin C, tau/Aβ42 + VEGF, tau/Aβ42 + KIM-1, tau/Aβ42 + PP) were combined with the 10 RBM analytes with the highest individual AUCs (indicated in Table 4). Because an independent validation cohort was not available for analysis, bootstrapping resampling with 100 iterations was performed to obtain relatively unbiased estimates of expected future performance of the ‘3-marker panels’ in predicting CDR 0 versus CDR>0 (Table 5). A number of the 3-marker panels demonstrated significantly improved AUCs compared to the corresponding 2-marker panels, with the best achieving AUCs of ∼.90 and sensitivities of ∼84% at 80% specificity (Table 5).

**Table 4:** ROC analyses.

AUC of Traditional Biomarkers			
**log A**β42	0.7552		
**log tau**	0.7830		
**log p-tau181**	0.7149		
**log tau/Aβ42**	0.8443		
**log p-tau181/Aβ42**	0.8065		

AUC of RBM Biomarkers: alone and in combination with traditional biomarkers			
	Marker	Marker+log tau/Aβ42	Marker+log p-tau181/Aβ42
**log α1A**	0.6296	0.8578	0.8234
**log ApoD**	0.6136	0.8489	0.8138
**log CKMB**	0.6106	0.8475	0.8118
**Cortisol**	0.6183	0.8510	0.8155
**log Cystatin C**	0.5965	0.8819 §	0.8468
**Eotaxin-3**	0.6448 §	0.8516	0.8202
**log FABP**	0.6163	0.8499	0.8080
**log FAS**	0.6689 §	0.8518	0.8209
**FASL**	0.6134	0.8479	0.8116
**log Fibrinogen**	0.6503 §	0.8564	0.8232
**GRO-α**	0.7024 §	0.8609	0.8305
**log HB-EGF**	0.5929	0.8445	0.8081
**log HCC-4**	0.6172	0.8596	0.8281
**log HGF**	0.5972	0.8458	0.8069
**log IGF-BP2**	0.6378	0.8462	0.8116
**IL-7**	0.6029	0.8508	0.8162
**log IL-10**	0.6075	0.8575	0.8215
**IL-17E**	0.5969	0.8487	0.8145
**log Insulin**	0.5406	0.8453	0.8077
**log IP-10**	0.5970	0.8460	0.8093
**KIM-1**	0.5894	0.8668 §	0.8343
**MCP-2**	0.6264	0.8554	0.8200
**log MIF**	0.6651 §	0.8455	0.8117
**log MIG**	0.6376	0.8544	0.8207
**log MMP-10**	0.6929 §	0.8518	0.8232
**log NT-proBNP**	0.6753 §	0.8562	0.8248
**log Osteopontin**	0.6050	0.8508	0.8100
**log PP**	0.6789 §	0.8644 §	0.8356
**log PAI-1**	0.6814 §	0.8587	0.8273
**log Resistin**	0.6218	0.8522	0.8211
**Sortilin**	0.6177	0.8444	0.8076
**log TNF RII**	0.6319	0.8447	0.8065
**TRAIL-R3**	0.6851 §	0.8523	0.8212
**Thrombomodulin**	0.6004	0.8503	0.8150
**Thrombopoietin**	0.5898	0.8465	0.8111
**TECK**	0.6371	0.8525	0.8190
**VEGF**	0.6146	0.8766 §	0.8441

To assess the ability of the markers to distinguish CDR>0 from CDR 0, ROC analyses were performed for each of the traditional biomarkers (Aβ42, tau, p-tau181 and the ratios tau/Aβ42 and p-tau181/Aβ42) and for the 37 RBM analytes with p<0.05 in the univariate analyses. Each traditional biomarker was then combined with each RBM analyte to identify ‘2-marker panels’ with improved AUCs. Among the traditional biomarkers, the ratios tau/Aβ42 and p-tau181/Aβ42 demonstrated the highest AUCs; additionally, combining these ratios with the RBM analytes consistently yielded 2-marker panels with AUCs higher than combinations of the individual traditional biomarkers (Aβ42, tau, p-tau181) with the RBM analytes. Thus, only the most promising 2-marker panels (those with tau/Aβ42 and p-tau181/Aβ42) are shown here. To determine whether combinations of three markers could yield a small panel with improved diagnostic accuracy, the four 2-marker panels with the highest AUCs were combined with the 10 RBM analytes with the highest individual AUCs (indicated by §, results in Table 5).

**Table 5:** ROC analyses of 3-marker panels.

Marker Panels	AUC	Stdev	95% CI	Sensitivity (at 80% specificity)	Stdev	95% CI	p-value	Stdev	95% CI
log tau/Aβ42 + log Cystatin C + TRAIL-R3	0.9014	0.0232	0.8969–0.9060	0.8367	0.0445	0.8280–0.8455	0.0299	0.0222	0.0255–0.0342
log tau/Aβ42 + log Cystatin C + log PAI-1	0.9063	0.0221	0.9020–0.9106	0.8470	0.0438	0.8384–0.8556	0.0283	0.0344	0.0215–0.0351
log tau/Aβ42 + log Cystatin C + log PP	0.9066	0.0203	0.9026–0.9106	0.8471	0.0400	0.8393–0.8550	0.0245	0.0319	0.0183–0.0307
log tau/Aβ42 + log Cystatin C + NT-proBNP	0.9041	0.0228	0.8996–0.9086	0.8422	0.0445	0.8335–0.8509	0.0287	0.0330	0.0223–0.0352
log tau/Aβ42 + log Cystatin C + log MMP-10	0.8987	0.0230	0.8942–0.9032	0.8317	0.0447	0.8230-0.8405	0.0647	0.0582	0.0533–0.0761
log tau/Aβ42 + log Cystatin C + log MIF	0.8964	0.0249	0.8915-0.9013	0.8272	0.0487	0.8177–0.8368	0.0699	0.0569	0.0588–0.0811
log tau/Aβ42 + log Cystatin C + GRO-α	0.9071	0.0218	0.9028–0.9113	0.8475	0.0412	0.8395–0.8556	0.0347	0.0410	0.0266–0.0427
log tau/Aβ42 + log Cystatin C + log Fibrinogen	0.9033	0.0219	0.8990–0.9075	0.8403	0.0429	0.8319–0.8487	0.0357	0.0502	0.0259–0.0455
log tau/Aβ42 + log Cystatin C + log FAS	0.9052	0.0220	0.9009–0.9095	0.8440	0.0425	0.8356–0.8523	0.0248	0.0248	0.0200–0.0297
log tau/Aβ42 + log Cystatin C + Eotaxin-3	0.9051	0.0219	0.9008–0.9094	0.8441	0.0427	0.8357–0.8524	0.0273	0.0350	0.0205–0.0342
log tau/Aβ42 + VEGF + TRAIL-R3	0.9004	0.0226	0.8960–0.9049	0.8347	0.0437	0.8262–0.8433	0.0208	0.0158	0.0177–0.0239
log tau/Aβ42 + VEGF + log PAI-1	0.9005	0.0225	0.8961–0.9049	0.8355	0.0445	0.8267–0.8442	0.0272	0.0320	0.0210–0.0335
log tau/Aβ42 + VEGF + log PP	0.9039	0.0215	0.8997–0.9081	0.8423	0.0422	0.8340–0.8506	0.0167	0.0250	0.0118–0.0216
log tau/Aβ42 + VEGF + NT-proBNP	0.9028	0.0224	0.8984–0.9072	0.8396	0.0439	0.8310–0.8482	0.0165	0.0207	0.0124–0.0205
log tau/Aβ42 + VEGF + log MMP-10	0.8947	0.0242	0.8900–0.8995	0.8241	0.0471	0.8149–0.8333	0.0534	0.0519	0.0432–0.0636
log tau/Aβ42 + VEGF + log MIF	0.8908	0.0261	0.8857–0.8959	0.8164	0.0506	0.8065–0.8264	0.0703	0.0570	0.0591–0.0815
log tau/Aβ42 + VEGF + GRO-α	0.9003	0.0238	0.8956–0.9049	0.8348	0.0452	0.8259–0.8436	0.0365	0.0371	0.0292–0.0437
log tau/Aβ42 + VEGF + log Fibrinogen	0.8988	0.0231	0.8943–0.9033	0.8317	0.0449	0.8229–0.8405	0.0327	0.0457	0.0237–0.0416
log tau/Aβ42 + VEGF + log FAS	0.9012	0.0232	0.8967–0.9058	0.8363	0.0445	0.8276–0.8451	0.0232	0.0248	0.0183–0.0281
log tau/Aβ42 + VEGF + Eotaxin-3	0.8991	0.0227	0.8947–0.9036	0.8325	0.0441	0.8239–0.8411	0.0293	0.0354	0.0224–0.0363
log tau/Aβ42 + KIM-1 + TRAIL-R3	0.8810	0.0256	0.8760–0.8860	0.7979	0.0486	0.7884–0.8075	0.1082	0.0747	0.0936–0.1229
log tau/Aβ42 + KIM-1 + log PAI-1	0.8866	0.0246	0.8818–0.8915	0.8087	0.0476	0.7993–0.8180	0.0614	0.0607	0.0495-0.0733
log tau/Aβ42 + KIM-1 + log PP	0.8905	0.0239	0.8858–0.8952	0.8162	0.0467	0.8070–0.8253	0.0357	0.0452	0.0269–0.0445
log tau/Aβ42 + KIM-1 + NT-proBNP	0.8821	0.0260	0.8770–0.8872	0.8001	0.0500	0.7903–0.8099	0.0926	0.0788	0.0772–0.1081
log tau/Aβ42 + KIM-1 + log MMP-10	0.8787	0.0270	0.8734–0.8840	0.7940	0.0511	0.7840–0.8040	0.1497	0.1015	0.1298–0.1696
log tau/Aβ42 + KIM-1 + log MIF	0.8775	0.0276	0.8721–0.8829	0.7918	0.0518	0.7816–0.8019	0.1478	0.0941	0.1294–0.1663
log tau/Aβ42 + KIM-1 + GRO-α	0.8897	0.0242	0.8850–0.8945	0.8153	0.0448	0.8065–0.8241	0.0513	0.0498	0.0416–0.0611
log tau/Aβ42 + KIM-1 + log Fibrinogen	0.8821	0.0267	0.8769–0.8874	0.8003	0.0507	0.7903–0.8102	0.0927	0.0809	0.0768–0.1085
log tau/Aβ42 + KIM-1 + log FAS	0.8806	0.0248	0.8757–0.8855	0.7973	0.0472	0.7881–0.8066	0.1157	0.0852	0.0990–0.1324
log tau/Aβ42 + KIM-1 + Eotaxin-3	0.8805	0.0264	0.8753–0.8857	0.7973	0.0498	0.7875-0.8071	0.1152	0.0943	0.0967–0.1337
log tau/Aβ42 + log PP + TRAIL-R3	0.8717	0.0249	0.8668–0.8766	0.7790	0.0488	0.7695–0.7886	0.2225	0.1023	0.2024–0.2425
log tau/Aβ42 + log PP + log PAI-1	0.8715	0.0250	0.8666–0.8764	0.7782	0.0498	0.7685–0.7880	0.2034	0.1052	0.1828–0.2240
log tau/Aβ42 + log PP + NT-proBNP	0.8723	0.0254	0.8674–0.8773	0.7806	0.0491	0.7710–0.7902	0.1705	0.1051	0.1499–0.1912
log tau/Aβ42 + log PP + log MMP-10	0.8702	0.0256	0.8652–0.8753	0.7761	0.0507	0.7662–0.7860	0.2394	0.1204	0.2158–0.2630
log tau/Aβ42 + log PP + log MIF	0.8685	0.0251	0.8635–0.8734	0.7723	0.0496	0.7625–0.7820	0.2909	0.1014	0.2711–0.3108
log tau/Aβ42 + log PP + GRO-α	0.8755	0.0250	0.8706–0.8804	0.7875	0.0472	0.7783–0.7968	0.1329	0.0908	0.1151–0.1507
log tau/Aβ42 + log PP + log Fibrinogen	0.8720	0.0255	0.8670–0.8769	0.7795	0.0498	0.7698–0.7893	0.1878	0.1160	0.1651–0.2106
log tau/Aβ42 + log PP + log FAS	0.8701	0.0244	0.8653–0.8749	0.7752	0.0487	0.7657–0.7847	0.2335	0.1091	0.2121–0.2548
log tau/Aβ42 + log PP + Eotaxin-3	0.8722	0.0245	0.8674–0.8770	0.7795	0.0487	0.7699–0.7890	0.1813	0.1087	0.1599–0.2026

AUC =  area under the curve; Stdev =  standard deviation; CI =  confidence interval.

Receiver operating characteristic (ROC) analyses assessed the ability of three marker panels to discriminate CDR 0 from CDR>0 participants. Averages of performance measures were taken over 100 iterations of the bootstrap. “p-value” assesses the difference between the three marker panel and the corresponding two marker panel (e.g. log tau/Aβ42 + log Cystatin C + TRAIL-R3 vs. log tau/Aβ42 + log Cystatin C).

**Figure 2:**
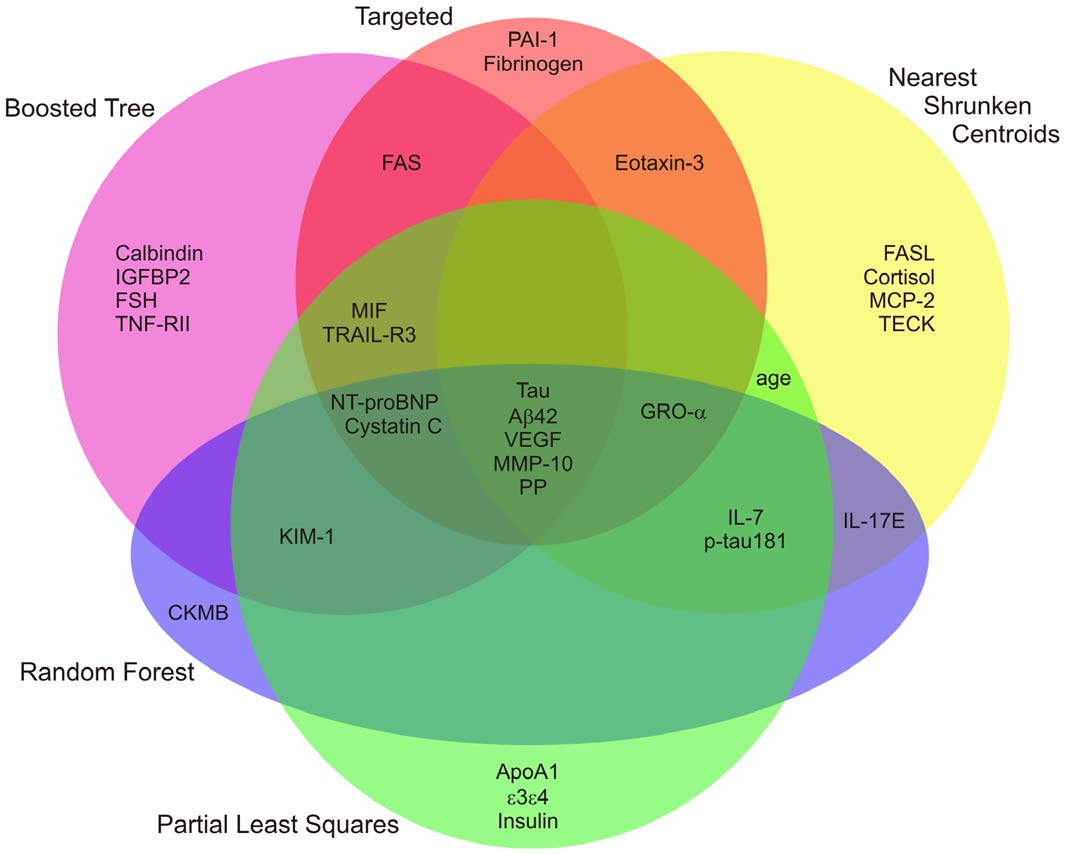
Venn diagram of the top 15 predictors for machine learning algorithms with a built-in importance measure. For the four models with a built-in importance statistic (i.e., Boosted Tree, Nearest Shrunken Centroids, Random Forests, and Partial Least Squares), there is considerable overlap in the top 15 predictors for each model. Additionally, nearly all of the markers found to best discriminate CDR 0 from CDR>0 participants in the more targeted ROC analyses (Table 5), as shown here (‘Targeted’), were also identified as the top predictors in the machine learning models.

Because AD is a complex, multifactorial disease and likely involves alterations in multiple biological pathways, it is possible that a larger panel of biomarkers encompassing various features of AD pathophysiology may be optimal for disease diagnosis. Thus, we utilized statistical machine learning algorithms, which are more amenable to potentially large numbers of analyte combinations and can identify highly complex nonlinear relationships, to discover whether groups of markers are capable of distinguishing very mildly/mildly demented (CDR 0.5 and 1 combined) from cognitively normal participants (CDR 0). A multi-pronged analytical approach including RF, PLS, SPLS, Boosted Tree, FDA, NB, NSC, LR, KNN, and SVM was used, as each approach has its own strengths and weaknesses. Models were fit with two sets of predictors: 1) traditional biomarkers, and 2) traditional biomarkers plus RBM analytes; additionally, age, gender, and ApoE genotype were included in all models. Model performance measures were based on cross-validation, in which the test set results were averaged from 200 splits of the data between training (80%) and test (20%) (Table 6). Using either traditional biomarkers or traditional biomarkers with RBM analytes, no model clearly out-performed the others; however, the RBM analytes appeared to contribute additional specificity to the biomarker panels (traditional: sensitivity 80.6–91.4%, specificity 42.4–56.6%; traditional+ RBM: sensitivity 79.1–93.2%, specificity 59.6–77.6%). This improvement is further reflected in the Youden Index, a single statistic that captures the performance of a diagnostic test and is a function of sensitivity and specificity, which was higher on average for the models fitted with traditional plus RBM analytes (traditional: 0.230–0.438; traditional+RBM: 0.401–0.621). Additionally, models fitted with traditional plus RBM analytes yielded mostly higher AUCs (traditional: 0.680–0.827; traditional+RBM: 0.754–0.868). For the four models with a built-in importance statistic (i.e., Boosted Tree, NSC, RF, and PLS) there was considerable overlap in the top 15 predictors for each model (Figure 2, Table 7). Importantly, nearly all of the markers found to best discriminate CDR 0 from CDR>0 participants in the more targeted ROC analyses (Table 5) were also identified as the top predictors in the machine learning models (Figure 2, Table 7), reconfirming the potential of these analytes as biomarkers for AD.

**Table 6:** Performance measures of machine learning algorithms in discriminating cognitively normal (CDR 0) from very mildly/mildly demented (CDR 0.5 and 1) participants.

	Traditional Biomarkers				Traditional + RBM Biomarkers			
Model	Sensitivity	Specificity	Youden Index	AUC	Sensitivity	Specificity	Youden Index	AUC
Boosted Tree	0.843	0.525	0.368	0.782	0.845	0.776	0.621	0.868
Flexible Discriminant Analysis	0.882	0.546	0.428	0.827	0.827	0.672	0.499	0.808
K-Nearest Neighbors	0.866	0.552	0.418	0.813	0.886	0.627	0.513	0.814
Logistic Regression	0.902	0.490	0.392	0.819	0.791	0.667	0.458	0.757
Naïve Bayes	0.898	0.492	0.390	0.799	0.802	0.599	0.401	0.754
Partial Least Squares	0.914	0.457	0.371	0.822	0.858	0.693	0.551	0.851
Sparse Partial Least Squares	0.914	0.457	0.371	0.822	0.858	0.694	0.552	0.851
Random Forests	0.872	0.566	0.438	0.810	0.932	0.596	0.528	0.866
Nearest Shrunken Centroids	0.882	0.527	0.409	0.805	0.833	0.643	0.476	0.802
Support Vector Machine	0.806	0.424	0.230	0.680	0.929	0.645	0.574	0.868

Ten statistical machine learning algorithms were used to determine groups of markers capable of distinguishing very mildly/mildly demented (CDR 0.5 and 1 combined) from cognitively normal participants (CDR 0). Models were fit with two sets of predictors: 1) traditional biomarkers, or 2) traditional biomarkers plus RBM analytes; additionally, age, gender, and ApoE4 allele status were included in all models. Model performance measures shown are based on cross-validation, in which the test set results were averaged from 200 splits of the data between training (80%) and test (20%).

**Table 7:** Top 15 predictors for machine learning algorithms with a built-in importance measure.

Predictor	Boosted Tree	Nearest Shrunken Centroids	Random Forests	Partial Least Squares
1	tau	Tau	Aβ42	Tau
2	Aβ42	Aβ42	tau	Aβ42
3	VEGF	p-tau181	MMP-10	VEGF
4	MMP-10	GRO-α	KIM-1	p-tau181
5	PP	VEGF	VEGF	GRO-α
6	KIM-1	Eotaxin-3	IL-7	PP
7	Cystatin C	Age	IL-17E	Cystatin C
8	Calbindin	PP	PP	NT-proBNP
9	NT-proBNP	Cortisol	NT-proBNP	MMP-10
10	MIF	MCP-2	TRAIL-R3	KIM-1
11	IGFBP-2	TECK	p-tau181	Apo A1
12	TRAIL-R3	MMP-10	Cystatin C	ε3ε4
13	FSH	IL-17E	MIF	IL-7
14	FAS	IL-7	GRO-α	Insulin
15	TNF RII	FASL	CKMB	Age

Ranking of the top 15 predictors for the four models with a built-in importance statistic demonstrates considerable overlap in the top predictors for each model. Furthermore, nearly all of the markers found to best discriminate CDR 0 from CDR>0 participants in the more targeted ROC analyses (Table 5) were also identified as the top predictors in the machine learning models, reconfirming their biomarker potential.

### Prognostic Utility of Candidate Biomarkers

Identifying individuals with AD neuropathology while they are still in the preclinical phase will be critically important, as disease-modifying therapies currently in development are likely to be most effective early in the disease process before significant synaptic and neuronal loss has occurred. Thus, we used univariate and multivariate Cox proportional hazards models to evaluate the ability of the analytes to predict risk of developing cognitive impairment (conversion from CDR 0 to CDR>0). Of the 215 CDR 0 subjects with at least one follow-up annual clinical assessment, 29 received a CDR>0 at follow-up, and thus were classified as “converters.” Analyte measurements were converted to standard Z-scores to allow for comparison of hazard ratios between the different analytes. Variables with p<0.15 in the univariate Cox analyses were considered for inclusion in the multivariate model; variables were retained in the final model if p<0.05. By univariate Cox analysis, calbindin (p = 0.0163), cortisol (p = 0.0688), HGF (p = 0.1364), MCP-2 (p = 0.0412), MIG (p = 0.0208), MIF (p = 0.0950), S100B (p = 0.1275), TNF RII (p = 0.0645), TRAIL-R3 (p = 0.0833), Aβ42 (p = <0.0001), tau (p = 0.0071), and p-tau181 (p = 0.0087) were selected for further investigation by multivariate analysis. The final multivariate model consisted of calbindin (HR = 1.750, p = 0.0063), 1/Aβ42 (HR = 2.454, p<0.0001), and age at LP (HR = 1.096, p = 0.0002), with an overall HR of 4.704 (Table 8). Although calbindin and tau both had p<0.05 in the univariate analysis, the significant correlation between the two (*r* = 0.476, p<0.0001) prohibited inclusion of both variables in the multivariate model. Therefore, a second multivariate model consisted of tau (HR = 1.467, p = 0.0262), 1/Aβ42 (HR = 2.247, p<0.0001), and age at LP (HR = 1.098, p = 0.0003), with an overall HR of 3.619 (Table 8). However, the higher HR of calbindin than of tau, and the higher overall HR and lower AIC of the first model support it as the better model.

**Table 8:** Cox proportional hazards models for predicting risk of developing cognitive impairment (conversion from CDR 0 to CDR>0).

A.	Marker	HR	95% CI	P	
	Log Calbindin	1.736	1.161–2.596	0.0072	
	Log 1/Aβ42	2.361	1.564–3.564	<0.0001	
	Age	1.094	1.043–1.147	0.0002	
	Gender	0.722	0.326–1.599	0.4216	

B.	Marker	HR	95% CI	P	
	Log Calbindin	1.752	1.176–2.609	0.0058	
	Log 1/Aβ42	2.485	1.655–3.731	<0.0001	
	Age	1.092	1.037–1.149	0.0008	
	ApoE4	0.847	0.355–2.025	0.7094	

C.	Marker	HR	95% CI	P	Overall HR 4.704
	Log Calbindin	1.750	1.172–2.613	0.0063	
	Log 1/Aβ42	2.454	1.637–3.679	<0.0001	
	Age	1.096	1.045–1.149	0.0002	

D.	Marker	HR	95% CI	P	
	Log Tau	1.462	1.039–2.057	0.0294	
	Log 1/Aβ42	2.221	1.477–3.339	0.0001	
	Age	1.096	1.041–1.154	0.0005	
	Gender	0.724	0.334–1.566	0.4113	

E.	Marker	HR	95% CI	P	Overall HR 3.610
	Log Tau	1.467	1.046–2.056	0.0262	
	Log 1/Aβ42	2.247	1.496–3.375	<0.0001	
	Age	1.098	1.043–1.156	0.0003	

Cox proportional hazards models were used to identify panels of biomarkers predictive of the risk of developing cognitive impairment (conversion from CDR 0 to CDR>0). Analyte measurements were converted to standard Z-scores to allow for comparison of hazard ratios between the different analytes. Variables with p<0.15 in the univariate Cox analyses were considered for inclusion in multivariate models; variables were retained in the final model if p<0.05. Because many of the analytes, including calbindin, demonstrated age and gender affects, both variables were entered into the multivariate models. However, as gender did not appear to contribute to the models (A, D), it was not included in the final panels (C, E). Similarly, apoE allelic status (E4+ vs. E4−) did not contribute to the models (B), and was not included in the final model (C). Although calbindin and tau both demonstrated p<0.05 in the univariate analyses, the significant correlation between the two (*r* = 0.476, p<0.0001) prohibited inclusion of both variables in the multivariate model. Therefore, a separate multivariate model that included tau was evaluated (D, E). The higher HR of calbindin than of tau, and the higher overall HR (4.704>3.610) and lower AIC (227.6<230.8) of the first model support it as the better model.

## Discussion

Biomarkers that can detect AD in its early stages and, importantly, predict future dementia will be invaluable for efficient clinical trial design and eventually patient care. This study identifies novel biomarkers that improve upon the ability of the best identified biomarkers to date to discriminate very mildly demented from cognitively normal participants, and identifies a novel biomarker with significant prognostic potential.

Using Luminex technology and a targeted multiplex panel, we identified 37 analytes (12 with Bonferroni correction) that are increased or decreased in the CSF of participants with early AD relative to cognitively normal controls. ROC analysis revealed that small combinations of a subset of these markers (cystatin C, VEGF, TRAIL-R3, PAI-1, PP, NT-proBNP, MMP-10, MIF, GRO-α, fibrinogen, FAS, and eotaxin-3) can enhance the ability of the best-performing of the traditional biomarkers, the tau/Aβ42 ratio, to discriminate CDR 0.5 and 1 from CDR 0 participants. Using alternative statistical strategies that are more amenable to the analysis of larger combinations of markers, multiple machine learning algorithms likewise showed that the novel biomarkers improved upon the diagnostic performance of the traditional biomarkers (Aβ42, tau, p-tau181). Importantly, nearly all of the markers found to best discriminate CDR 0 from CDR 0.5 and 1 participants in the more targeted ROC analyses were also identified as the top predictors in the machine learning models that contain a built-in importance statistic (10 of 12 markers). Thus, the potential of these analytes as biomarkers for AD is supported by alternative statistical approaches that yielded similar results. Further supporting these results is a recent report of the application of a smaller RBM Discovery MAP panel to a smaller cohort of AD, MCI, and control subjects [18]; this study identified a number of the same analytes as being differentially expressed in AD CSF as compared to control CSF and, although using different analytical approaches, included VEGF, TRAIL-R3, and eotaxin-3, in ‘combined’ models of novel and traditional biomarkers.

It is important to note that while the models used in our study suggest diagnostic value of the novel biomarkers, other combinations of these markers may be optimal; it will be of interest in future studies to validate the results of this discovery study in additional cohorts and to determine whether alternative combinations of these markers may demonstrate improved performance. The levels of at least 7 of the novel biomarkers have been evaluated in AD subjects in other studies: no change was observed in plasma PAI-1 levels [44]; in agreement with our findings, two studies have reported increased CSF MIF in AD and MCI subjects [45], [46]; also consistent with our findings, increased fibrinogen levels have been observed in AD and MCI CSF [47] and in AD plasma [48], and increased plasma levels have been associated with an increased risk of future dementia [49]; results have been mixed regarding CSF FAS levels in AD [50], [51]; AD plasma/serum VEGF levels have been reported to be unchanged [52], [53], decreased [54], and increased [55], while CSF levels have been reported to be unchanged [56] or increased [57]; no change in CSF or serum levels of TNF RII in AD has been reported [58]; cystatin C findings have been inconsistent, with reports of serum/plasma levels unchanged [59], increased in AD [60] or in those who later develop AD [61], and decreased [62] or decreased levels associated with increased risk of future AD [63], while CSF levels have been reported to be unchanged [59], [64], decreased [65], or increased [21]. These inconsistent results may be due in part to the existence of a truncated form of cystatin C, which was found to be increased in AD CSF, while the full length protein was decreased [20], [21].

Furthermore, the potential involvement of each marker in AD pathophysiology necessitates investigation. The candidate biomarkers identified in the ROC and machine learning portions of this study belong to a wide variety of functional classes and pathways, including tissue remodeling and angiogenesis (MMP-10, VEGF), regulation of apoptosis (TRAIL-R3, FAS), neutrophil, eosinophil, and/or basophil chemotaxis (GRO-α, eotaxin-3), blood coagulation (Fibrinogen, PAI-1), intravascular volume homeostasis (NT-proBNP), and gastrointestinal and pancreatic secretions (PP). In addition, a number of molecules involved in inflammatory pathways were identified in the machine learning models (IL-7, IL-17E, TNF RII, MCP-2, FASL, MIF). The association of several of the candidate biomarkers with AD pathophysiology has already been probed, most notably for cystatin C, which appears to play a role in preventing Aβ oligomerization and amyloidogenesis [66]–[70], and to a lesser extent for PAI-1 [71]–[73], MIF [45], [74], fibrinogen [75], [76], FAS and FASL [77]–[80], VEGF [81]–[83], and TNF RII [84]–[86].

It will be important in future studies to assess each candidate biomarker's value in diagnosis in independent sample sets when combined with other existing biomarkers or imaging tools. The existing gold standard validated biomarkers include CSF tau, p-tau181, and amyloid imaging, which differ between control and AD populations and mark underlying AD pathology [4], [6], [31], [32]. Additionally, to follow up on these biomarker candidates, their ability to discriminate AD from other causes of dementia needs to be examined; indeed, several of these markers have already shown promise for distinguishing AD from frontotemporal lobar degeneration (cystatin C [20], eotaxin-3 [18], and HGF [18]). Incorporation of such markers into a biomarker panel may improve diagnostic specificity. Beyond their clinical use, these markers may have great value in the design of and enrollment in trials of disease-modifying therapies. By enrolling only subjects with lower or higher values of a particular marker (or panels of markers) indicative of AD, and excluding potential subjects with intermediate or ‘overlap’ values, one might provide greater diagnostic certainty than is possible through clinical evaluation alone. This is especially relevant for the design and evaluation of primary prevention trials in cognitively normal cohorts. Enriching study populations for subjects displaying certain biomarker levels may result in studies of greater efficacy, translating to reduced cost and duration.

This study also suggests a novel biomarker, CSF calbindin, that can predict risk of future dementia in individuals who are still cognitively normal. Previous studies have shown that Aβ42, tau, YKL-40 (an astrocyte marker), and the ratios tau/Aβ42 and YKL-40/Aβ42 can predict subsequent cognitive decline in non-demented cohorts [7], [15], [87]. Using multivariate Cox proportional hazards models to determine the best combination of biomarkers for prognosis, we show here that a panel of markers consisting of calbindin, Aβ42, and age has predictive value comparable to, if not better than, a second panel consisting of tau, Aβ42, and age. Tissue culture studies have shown that increased expression of calbindin, a calcium binding protein present in central and peripheral nervous system neurons, correlates with increased resistance to cell death triggered by a variety of causes, including exposure to excitatory amino acids, ischemic injury, and Aβ [88]–[91]. Decreases in calbindin protein and mRNA levels [92] and number of calbindin-immunopositive neurons [93]–[95] have been observed in AD brains compared to controls. Further suggesting there may be a role for calbindin in AD pathophysiology is the large body of literature demonstrating that increased oxidative stress and altered calcium homeostasis appear to be interrelated mechanisms in AD pathogenesis. Interestingly, although not quite reaching statistical significance, we found that CSF calbindin levels trended higher in the very mildly/mildly demented group (p = .0660; CDR 0 =  145.9 ng/mL, CDR>0 =  157.4 ng/mL), suggesting that perhaps degenerating neurons release calbindin into the CSF. The immunohistochemical findings of a small study of 6 AD brains suggesting that calbindin-immunopositive neurons are relatively preserved in cases with moderate amyloid plaque and neurofibrillary content but are lost in more severe cases [94] prompts the question of whether CSF calbindin levels would be more significantly elevated in more severely demented individuals. Further studies are needed to confirm the prognostic potential of CSF calbindin, to determine if other complementary fluid or imaging biomarkers may improve upon its performance, and to more definitively elucidate its role in AD pathophysiology. As with the candidate diagnostic biomarkers, CSF calbindin may have value for clinical trial design by allowing for the selective enrollment of individuals who are at greater risk of developing cognitive impairment, resulting in clinical trials of shorter duration and reduced cost.

## Supporting Information

Table S1 **Means and standard deviations of the 125 RBM analytes and traditional biomarkers.** The means and standard deviations of the 125 measurable RBM analytes and the traditional biomarkers are provided.
(DOC)

Table S2 **ANCOVA: Age and gender interactions.** As shown in Table 2, the mean concentrations of 37 CSF RBM analytes were found to differ between cognitively normal (CDR 0) and very mildly/mildly demented (CDR 0.5 and 1) participants by analysis of covariance (ANCOVA) adjusting for age and gender (p<0.05). ANCOVA showed that a number of these analytes demonstrated significant interactions with age or gender, as shown here.
(DOC)
