# Supplementary material for: Multiplexed Immunoassay Panel Identifies Novel CSF Biomarkers for Alzheimer's Disease Diagnosis and Prognosis
Source: PLoS One. 2011 Apr 19;6(4):e18850. doi: 10.1371/journal.pone.0018850 (PMC3079734; doi:10.1371/journal.pone.0018850)
Supplement: Table S1 — Means and standard deviations of the 125 RBM analytes and traditional biomarkers. The means and standard deviations of the 125 measurable RBM analytes and the traditional biomarkers are provided. (DOC) [file pone.0018850.s001.doc]

**Table S1. Means and standard deviations of the 125 RBM analytes and traditional biomarkers.**

| **Marker** | **Log transformed?** | **Mean CDR 0** | **Stdev** | **Mean CDR>0** | **Stdev** |
| --- | --- | --- | --- | --- | --- |
| Adiponectin (ug/mL) | yes | -2.287 | 0.282 | -2.216 | 0.302 |
| Adrenocorticotropic Hormone (ACTH) (ng/mL) | yes | -0.672 | 0.116 | -0.656 | 0.125 |
| Alpha-1-Antichymotrypsin (ACT) (ug/mL) | yes | 0.574 | 0.158 | 0.623 | 0.162 |
| Alpha-1-Antitrypsin (mg/mL) | yes | -2.325 | 0.133 | -2.261 | 0.140 |
| Alpha-1-Microglobulin (A1M) (ug/mL) | yes | -1.298 | 0.140 | -1.225 | 0.195 |
| Alpha-2-Macroglobulin (A2M) (mg/mL) | yes | -2.626 | 0.153 | -2.589 | 0.155 |
| Angiopoietin-2 (ANG-2) (ng/mL) | yes | 0.278 | 0.144 | 0.302 | 0.147 |
| Angiotensin-Converting Enzyme (ACE) (ng/mL) | yes | 0.471 | 0.181 | 0.469 | 0.167 |
| Angiotensinogen (AGT) (ng/mL) | yes | 1.742 | 0.389 | 1.809 | 0.396 |
| Apolipoprotein A-I (ApoA1) (mg/mL) | yes | -3.247 | 0.191 | -3.257 | 0.192 |
| Apolipoprotein A-II (ApoA2) (ng/mL) | yes | -0.287 | 0.216 | -0.265 | 0.209 |
| Apolipoprotein A-IV (ApoAIV) (ug/mL) | yes | -0.810 | 0.168 | -0.800 | 0.162 |
| Apolipoprotein B (ApoB) (ug/mL) | yes | -1.426 | 0.238 | -1.350 | 0.266 |
| Apolipoprotein C-I (ApoC1) (ng/mL) | yes | -0.691 | 0.186 | -0.687 | 0.194 |
| Apolipoprotein C-III (ApoC3) (ug/mL) | yes | -1.099 | 0.193 | -1.049 | 0.193 |
| Apolipoprotein D (ApoD) (ug/mL) | yes | 0.604 | 0.144 | 0.667 | 0.166 |
| Apolipoprotein E (ApoE) (ug/mL) | yes | 0.802 | 0.162 | 0.795 | 0.187 |
| Apolipoprotein H (ApoH) (ug/mL) | yes | -0.180 | 0.194 | -0.122 | 0.181 |
| Apolipoprotein J (ApoJ) (ug/mL) | yes | 1.235 | 0.128 | 1.279 | 0.143 |
| Apolipoprotein (a) (ug/mL) | yes | -1.943 | 0.478 | -1.846 | 0.505 |
| AXL Receptor Tyrosine Kinase (ng/mL) | yes | 0.094 | 0.186 | 0.124 | 0.150 |
| Aβ42 (pg/mL) | no | 606.901 | 233.707 | 411.181 | 207.254 |
| B Lymphocyte Chemoattractant (BLC) (pg/mL) | yes | 0.615 | 0.155 | 0.667 | 0.133 |
| Beta-2-Microglobulin (B2M) (ug/mL) | yes | 0.067 | 0.127 | 0.080 | 0.135 |
| Betacellulin (pg/mL) | no | 51.480 | 10.940 | 50.600 | 10.525 |
| Bone Morphogenetic Protein 6 (BMP-6) (ng/mL) | yes | -1.063 | 0.230 | -1.060 | 0.228 |
| Calbindin (ng/mL) | no | 147.662 | 46.828 | 161.130 | 61.241 |
| Calcitonin (pg/mL) | yes | 0.731 | 0.369 | 0.742 | 0.412 |
| CD40 (ng/mL) | yes | -0.633 | 0.120 | -0.646 | 0.145 |
| CD5 Antigen-like (CD5L) (ng/mL) | yes | -0.039 | 0.198 | 0.015 | 0.212 |
| Chromogranin A (ng/mL) | no | 145.692 | 30.712 | 146.995 | 30.044 |
| Complement C3 (mg/mL) | yes | -2.528 | 0.190 | -2.449 | 0.198 |
| Complement Factor H (ug/mL) | no | 9.585 | 4.907 | 10.059 | 4.883 |
| Connective Tissue Growth Factor (CTGF) (ng/mL) | no | 2.185 | 0.462 | 2.236 | 0.450 |
| Cortisol (ng/mL) | no | 11.166 | 3.692 | 12.887 | 4.365 |
| C-Reactive Protein (CRP) (ug/mL) | yes | -2.540 | 0.543 | -2.578 | 0.525 |
| Creatine Kinase-MB (CKMB) (ng/mL) | yes | -1.575 | 0.273 | -1.680 | 0.258 |
| Cystatin C (ng/mL) | yes | 3.744 | 0.174 | 3.684 | 0.173 |
| Eotaxin-3 (pg/mL) | no | 55.327 | 15.961 | 63.678 | 15.832 |
| Epidermal Growth Factor Receptor (EGFR) (ng/mL) | no | 0.450 | 0.145 | 0.461 | 0.137 |
| Epithelial-Derived Neutrophil-Activating Protein 78 (ENA-78) (ng/mL) | no | 0.010 | 0.003 | 0.009 | 0.003 |
| Extracellular Newly Identified Receptor for Advanced Glycation Endproducts (ENRAGE) (ng/mL) | yes | -1.565 | 0.405 | -1.572 | 0.410 |
| FAS (ng/mL) | yes | -0.252 | 0.123 | -0.173 | 0.133 |
| Fatty Acid Binding Protein (FABP) (ng/mL) | yes | 0.467 | 0.260 | 0.581 | 0.289 |
| Fatty Acid Synthase Ligand (FASL) (pg/mL) | no | 4.780 | 1.632 | 5.495 | 1.867 |
| Ferritin (ng/mL) | no | 5.657 | 1.773 | 6.167 | 2.101 |
| Fetuin-A (ug/mL) | yes | 0.570 | 0.171 | 0.622 | 0.154 |
| Fibrinogen (mg/mL) | yes | -3.230 | 0.258 | -3.092 | 0.248 |
| Follicle-Stimulating Hormone (FSH) (ng/mL) | no | 0.264 | 0.180 | 0.256 | 0.192 |
| Gamma-Interferon-Induced Monokine (MIG) (pg/mL) | yes | 2.602 | 0.319 | 2.758 | 0.307 |
| Glutathione S-Transferase alpha (GST) (ng/mL) | yes | 0.523 | 0.122 | 0.527 | 0.086 |
| Growth-Regulated alpha protein (GRO-α) (pg/mL) | no | 18.304 | 3.924 | 22.439 | 6.721 |
| Hemofiltrate cysteine-cysteine chemokine (HCC-4) (ng/mL) | yes | -1.538 | 0.163 | -1.470 | 0.158 |
| Heparin-binding epidermal growth factor-like growth factor (HB-EGF) (pg/mL) | yes | 1.399 | 0.182 | 1.458 | 0.173 |
| Hepatocyte Growth Factor (HGF) (ng/mL) | yes | 0.071 | 0.128 | 0.115 | 0.127 |
| Immunoglobulin A (IgA) (mg/mL) | yes | -2.675 | 0.308 | -2.580 | 0.299 |
| Immunoglobulin M (IgM) (mg/mL) | yes | -3.959 | 0.312 | -3.975 | 0.313 |
| Insulin (uIU/mL) | yes | -0.675 | 0.236 | -0.711 | 0.264 |
| Insulin-like Growth Factor-Binding Protein 2 (IGFBP-2) (ng/mL) | yes | 2.292 | 0.085 | 2.337 | 0.096 |
| Intercellular Adhesion Molecule 1 (ICAM-1) (ng/mL) | no | 0.538 | 0.253 | 0.563 | 0.244 |
| Interferon gamma Induced Protein 10 (IP-10) (pg/mL) | yes | 2.470 | 0.222 | 2.550 | 0.238 |
| Interleukin-10 (IL-10 ) (pg/mL) | yes | 0.048 | 0.175 | 0.111 | 0.149 |
| Interleukin-11 (IL-11) (pg/mL) | yes | 1.423 | 0.315 | 1.398 | 0.356 |
| Interleukin-13 (IL-13) (pg/mL) | no | 26.652 | 3.215 | 26.192 | 2.602 |
| Interleukin-16 (IL-16) (pg/mL) | no | 10.597 | 4.542 | 10.529 | 4.286 |
| Interleukin-17E (IL-17E) (pg/mL) | no | 8.603 | 2.666 | 7.793 | 1.948 |
| Interleukin-1alpha (IL-1α) (pg/mL) | yes | -3.271 | 0.174 | -3.252 | 0.178 |
| Interleukin-3 (IL-3) (ng/mL) | yes | -1.711 | 0.219 | -1.733 | 0.218 |
| Interleukin-4 (IL-4) (pg/mL) | yes | 0.774 | 0.218 | 0.748 | 0.232 |
| Interleukin-5 (IL-5) (pg/mL) | yes | 0.091 | 0.200 | 0.071 | 0.187 |
| Interleukin-6 (IL-6) (pg/mL) | no | 0.976 | 0.532 | 1.024 | 0.676 |
| Interleukin-6 Receptor (IL-6R) (ng/mL) | no | 1.129 | 0.339 | 1.124 | 0.362 |
| Interleukin-7 (IL-7) (pg/mL) | no | 12.232 | 7.621 | 9.677 | 6.160 |
| Interleukin-8 (IL-8) (pg/mL) | yes | 1.657 | 0.100 | 1.689 | 0.121 |
| Kidney Injury Molecule-1 (KIM-1) (ng/mL) | no | 0.079 | 0.014 | 0.083 | 0.011 |
| Lectin-Like Oxidized LDL Receptor 1 (LOX-1) (ng/mL) | yes | 0.550 | 0.174 | 0.550 | 0.193 |
| Leptin (ng/mL) | no | 0.121 | 0.075 | 0.106 | 0.069 |
| Macrophage Inflammatory Protein-1 alpha (MIP-1α) (pg/mL) | no | 7.668 | 2.361 | 8.318 | 1.993 |
| Macrophage Inflammatory Protein-1 beta (MIP-1β) (pg/mL) | yes | 1.211 | 0.166 | 1.244 | 0.174 |
| Macrophage Migration Inhibitory Factor (MIF) (ng/mL) | yes | -0.838 | 0.147 | -0.755 | 0.126 |
| Matrix Metalloproteinase-10 (MMP-10) (ng/mL) | yes | -1.618 | 0.180 | -1.487 | 0.188 |
| Matrix Metalloproteinase-2 (MMP-2) (ng/mL) | no | 5.579 | 1.857 | 5.287 | 1.773 |
| Matrix Metalloproteinase-3 (MMP-3) (ng/mL) | yes | -1.082 | 0.245 | -1.027 | 0.256 |
| Matrix Metalloproteinase-7 (MMP-7) (ng/mL) | yes | -0.917 | 0.280 | -0.805 | 0.230 |
| Monocyte Chemotactic Protein 1 (MCP-1) (pg/mL) | no | 676.050 | 181.842 | 703.495 | 178.258 |
| Monocyte Chemotactic Protein 2 (MCP-2) (pg/mL) | no | 3.966 | 1.397 | 4.670 | 1.680 |
| Myoglobin (ng/mL) | yes | -0.610 | 0.444 | -0.508 | 0.424 |
| Neuronal Cell Adhesion Molecule (NrCAM) (ng/mL) | yes | 1.892 | 0.252 | 1.879 | 0.242 |
| N-terminal pro-brain natriuretic peptide (NT-proBNP) (pg/mL) | yes | 1.943 | 0.156 | 2.046 | 0.164 |
| Osteopontin (ng/mL) | yes | 2.241 | 0.170 | 2.306 | 0.176 |
| Pancreatic Polypeptide (PP) (pg/mL) | yes | -0.057 | 0.295 | 0.150 | 0.333 |
| Peptide YY (PYY) (pg/mL) | yes | 1.306 | 0.125 | 1.307 | 0.125 |
| Placenta Growth Factor (PLGF) (pg/mL) | yes | 1.680 | 0.175 | 1.742 | 0.182 |
| Plasminogen Activator Inhibitor 1 (PAI-1) (ng/mL) | yes | 0.004 | 0.167 | 0.128 | 0.204 |
| Pregnancy-Associated Plasma Protein A (PAPP-A) (mlU/mL) | yes | -1.839 | 0.145 | -1.842 | 0.143 |
| Prolactin (ng/mL) | yes | 0.017 | 0.141 | 0.036 | 0.117 |
| Prostatic Acid Phosphatase (PAP) (ng/mL) | no | 0.025 | 0.008 | 0.027 | 0.010 |
| Protein S (ug/mL) | yes | -0.701 | 0.081 | -0.675 | 0.088 |
| p-tau181 (pg/mL) | no | 56.315 | 25.164 | 82.976 | 42.757 |
| Pulmonary and Activation-Regulated Chemokine (PARC) (ng/mL) | yes | -0.666 | 0.202 | -0.601 | 0.174 |
| Regulated on Activation, Normal T Expressed and Secreted (RANTES) (ng/mL) | yes | -2.841 | 0.142 | -2.793 | 0.148 |
| Resistin (ng/mL) | yes | -1.599 | 0.212 | -1.493 | 0.266 |
| S100B (ng/mL) | yes | 0.442 | 0.111 | 0.478 | 0.106 |
| Serum Amyloid P-Component (SAP) (ug/mL) | yes | -2.623 | 0.247 | -2.598 | 0.249 |
| Serum Glutamic Oxaloacetic Transaminase (SGOT) (ug/mL) | no | 0.693 | 0.281 | 0.722 | 0.209 |
| Sex Hormone-Binding Globulin (SHBG) (nmol/L) | yes | -1.104 | 0.250 | -1.064 | 0.243 |
| Sortilin (ng/mL) | no | 6.327 | 1.499 | 6.962 | 1.497 |
| Stem Cell Factor (SCF) (pg/mL) | yes | 1.432 | 0.158 | 1.423 | 0.147 |
| Superoxide Dismutase 1 (SOD-1) (ng/mL) | yes | 2.305 | 0.167 | 2.340 | 0.165 |
| T Lymphocyte-Secreted Protein I-309 (pg/mL) | yes | 1.269 | 0.157 | 1.316 | 0.176 |
| Tamm-Horsfall Urinary Glycoprotein (THP) (ug/mL) | yes | -3.978 | 0.207 | -3.951 | 0.202 |
| Tau (pg/mL) | no | 314.795 | 168.881 | 549.960 | 272.802 |
| Thrombomodulin (ng/mL) | no | 0.167 | 0.054 | 0.187 | 0.060 |
| Thrombopoietin (ng/mL) | no | 0.419 | 0.154 | 0.371 | 0.142 |
| Thymus-Expressed Chemokine (TECK) (ng/mL) | no | 6.295 | 1.323 | 6.958 | 1.350 |
| Thyroid-Stimulating Hormone (TSH) (uIU/mL) | yes | -1.883 | 0.317 | -1.872 | 0.301 |
| Thyroxine Binding Globulin (TBG) (ug/mL) | yes | -0.652 | 0.178 | -0.615 | 0.168 |
| Tissue Factor (TF) (ng/mL) | yes | 0.502 | 0.223 | 0.511 | 0.222 |
| Tissue Inhibitor of Metalloproteinases 1 (TIMP-1) (ng/mL) | no | 1.656 | 0.107 | 1.688 | 0.118 |
| TNF-Related Apoptosis-Inducing Ligand Receptor 3 (TRAIL-R3) (ng/mL) | no | 0.547 | 0.141 | 0.651 | 0.164 |
| Transferrin (mg/dL) | yes | 1.260 | 0.123 | 1.267 | 0.119 |
| Transforming Growth Factor alpha (TGF-α) (pg/mL) | no | 54.056 | 14.281 | 56.309 | 13.495 |
| Transthyretin (mg/dL) | no | 17.635 | 2.464 | 17.132 | 2.282 |
| Trefoil Factor 3 (TFF3) (ug/mL) | yes | -1.696 | 0.144 | -1.675 | 0.158 |
| Tumor necrosis factor-a receptor 2 (TNF RII) (ng/mL) | yes | -0.280 | 0.140 | -0.210 | 0.155 |
| Vascular Cell Adhesion Molecule-1 (VCAM-1) (ng/mL) | yes | 1.154 | 0.129 | 1.186 | 0.160 |
| Vascular Endothelial Growth Factor (VEGF) (pg/mL) | no | 437.835 | 130.622 | 386.011 | 120.754 |
| Vitronectin (ug/mL) | yes | -0.120 | 0.152 | -0.123 | 0.136 |
| von Willebrand Factor (vWF) (ug/mL) | yes | -1.717 | 0.166 | -1.677 | 0.170 |
