# Supplementary material for: Multiplexed Immunoassay Panel Identifies Novel CSF Biomarkers for Alzheimer's Disease Diagnosis and Prognosis
Source: PLoS One. 2011 Apr 19;6(4):e18850. doi: 10.1371/journal.pone.0018850 (PMC3079734; doi:10.1371/journal.pone.0018850)
Supplement: Table S2 — ANCOVA: Age and gender interactions. As shown in Table 2, the mean concentrations of 37 CSF RBM analytes were found to differ between cognitively normal (CDR 0) and very mildly/mildly demented (CDR 0.5 and 1) participants by analysis of covariance (ANCOVA) adjusting for age and gender (p<0.05). ANCOVA showed that a number of these analytes demonstrated significant interactions with age or gender, as shown here. (DOC) [file pone.0018850.s002.doc]

**Table S2. ANCOVA:** Age and gender interactions.

| **Marker** | **p value - Age** | **p value - Gender** |
| --- | --- | --- |
| Aβ42 (pg/mL) | 0.1039 | 0.3888 |
| Tau (pg/mL) | <0.0001 | 0.1236 |
| p-tau181 (pg/mL) | 0.0019 | 0.1575 |
| Growth-Regulated alpha protein (GRO-α) (pg/mL) | 0.0005 | 0.0487 |
| Log Matrix Metalloproteinase-10 (MMP-10) (pg/mL) | <0.0001 | 0.0281 |
| Log N-terminal pro-brain natriuretic peptide (NT-proBNP) (pg/mL) | <0.0001 | 0.0015 |
| Log Plasminogen Activator Inhibitor 1 (PAI-1) (ng/mL) | <0.0001 | 0.0006 |
| TNF-Related Apoptosis-Inducing Ligand Receptor 3 (TRAIL-R3) (ng/mL) | <0.0001 | 0.6402 |
| Vascular Endothelial Growth Factor (VEGF) (pg/mL) | 0.0020 | 0.7080 |
| Log Pancreatic Polypeptide (PP) (pg/mL) | <0.0001 | 0.0003 |
| Log FAS (ng/mL) | <0.0001 | 0.0012 |
| Log Macrophage Migration Inhibitory Factor (MIF) (ng/mL) | <0.0001 | 0.7029 |
| Interleukin-7 (IL-7) (pg/mL) | 0.0917 | 0.0124 |
| Log Cystatin C (ng/mL) | 0.0222 | 0.5164 |
| Thrombopoietin (ng/mL) | 0.0870 | 0.0131 |
| Sortilin (ng/mL) | 0.1747 | 0.4778 |
| Monocyte Chemotactic Protein 2 (MCP-2) (pg/mL) | 0.0204 | 0.1344 |
| Log Fibrinogen (ug/mL) | <0.0001 | <0.0001 |
| Log Creatine Kinase-MB (CKMB) (pg/mL) | 0.7073 | 0.9453 |
| Cortisol (ng/mL) | 0.0022 | 0.4898 |
| Thymus-Expressed Chemokine (TECK) (ng/mL) | <0.0001 | 0.0039 |
| Eotaxin-3 (pg/mL) | <0.0001 | 0.0013 |
| Interleukin-17E (IL-17E) (pg/mL) | 0.3400 | 0.8994 |
| Kidney Injury Molecule-1 (KIM-1) (pg/mL) | 0.1599 | 0.7826 |
| Log Heparin-binding epidermal growth factor-like growth factor (HB-EGF) (pg/mL) | 0.7232 | 0.8371 |
| Log Osteopontin (ng/mL) | 0.0051 | 0.0410 |
| Log α-1-Antitrypsin (ug/mL) | 0.0001 | <0.0001 |
| Fatty Acid Synthase Ligand (FASL) (pg/mL) | 0.0022 | 0.2526 |
| Log Insulin-like Growth Factor-Binding Protein 2 (IGFBP-2) (ng/mL) | <0.0001 | 0.0059 |
| Log Interleukin-10 (IL-10) (pg/mL) | 0.5571 | 0.0035 |
| Log Tumor necrosis factor-a receptor 2 (TNF RII) (ng/mL) | <0.0001 | 0.5670 |
| Log Resistin (pg/mL) | <0.0001 | 0.0046 |
| Log Fatty Acid Binding Protein (FABP) (ng/mL) | <0.0001 | 0.1413 |
| Log Apolipoprotein D (ApoD) (ug/mL) | 0.0018 | <0.0001 |
| Log Hepatocyte Growth Factor (HGF) (ng/mL) | 0.0006 | 0.5059 |
| Log Insulin (uIU/mL) | 0.0284 | 0.0002 |
| Log Hemofiltrate cysteine-cysteine chemokine (HCC-4) (pg/mL) | 0.0002 | <0.0001 |
| Log Interferon gamma Induced Protein 10 (IP-10) (pg/mL) | 0.0003 | 0.8961 |
| Log Gamma-Interferon-Induced Monokine (MIG) (pg/mL) | <0.0001 | 0.8703 |
| Thrombomodulin (ng/mL) | 0.0063 | 0.0009 |
